# Ten things you should know about transposable elements

**DOI:** 10.1186/s13059-018-1577-z

**Published:** 2018-11-19

**Authors:** Guillaume Bourque, Kathleen H. Burns, Mary Gehring, Vera Gorbunova, Andrei Seluanov, Molly Hammell, Michaël Imbeault, Zsuzsanna Izsvák, Henry L. Levin, Todd S. Macfarlan, Dixie L. Mager, Cédric Feschotte

**Affiliations:** 1 Department of Human Genetics, McGill University, Montréal, Québec H3A 0G1 Canada; 2 Canadian Center for Computational Genomics, McGill University, Montréal, Québec H3A 0G1 Canada; 3 Department of Pathology, Johns Hopkins University School of Medicine, Baltimore, MD 21205 USA; 4 Whitehead Institute for Biomedical Research and Department of Biology, Massachusetts Institute of Technology, Cambridge, MA 02142 USA; 5 Department of Biology, University of Rochester, Rochester, NY 14627 USA; 6 Cold Spring Harbor Laboratory, Cold Spring Harbor, NY 11724 USA; 7 Department of Genetics, University of Cambridge, Cambridge, CB2 3EH UK; 8 Max Delbrück Center for Molecular Medicine in the Helmholtz Association, 13125 Berlin, Germany; 9 The Eunice Kennedy Shriver National Institute of Child Health and Human Development, The National Institutes of Health, Bethesda, Maryland USA; 10 Terry Fox Laboratory, British Columbia Cancer Agency and Department of Medical Genetics, University of BC, Vancouver, BC V5Z1L3 Canada; 11 Department of Molecular Biology and Genetics, Cornell University, Ithaca, NY 14850 USA

## Abstract

Transposable elements (TEs) are major components of eukaryotic genomes. However, the extent of their impact on genome evolution, function, and disease remain a matter of intense interrogation. The rise of genomics and large-scale functional assays has shed new light on the multi-faceted activities of TEs and implies that they should no longer be marginalized. Here, we introduce the fundamental properties of TEs and their complex interactions with their cellular environment, which are crucial to understanding their impact and manifold consequences for organismal biology. While we draw examples primarily from mammalian systems, the core concepts outlined here are relevant to a broad range of organisms.

## Transposable elements come in many different forms and shapes

**Fig. 1:**
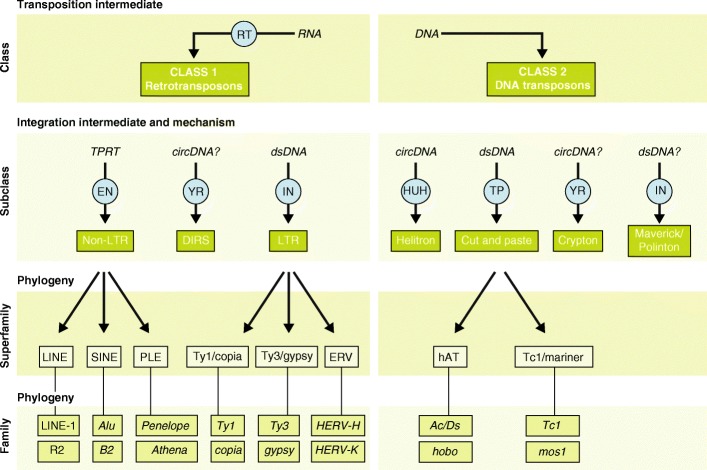
Classification of eukaryotic transposable elements. Schematic and examples showing the key features and relationships between TE classes, subclasses, superfamilies, and families. Blue circles represent TE-encoded enzymes. circDNA circular DNA intermediate, DIRS Dictyostelium repetitive sequence, dsDNA linear double-stranded DNA intermediate, EN endonuclease, IN integrase, PLEs Penelope-like elements, HUH, Rep/Helicase protein with HUH endonuclease activity, RT reverse transcriptase, TP transposase, TPRT target primed reverse transcription, YR tyrosine recombinase (for other abbreviations, see text)

Transposable elements (TEs) are DNA sequences that have the ability to change their position within a genome. As a result of their deep evolutionary origins and continuous diversification, TEs come in a bewildering variety of forms and shapes (Fig. 1). TEs can be divided into two major classes based on their mechanism of transposition, and each class can be subdivided into subclasses based on the mechanism of chromosomal integration. Class 1 elements, also known as retrotransposons, mobilize through a ‘copy-and-paste’ mechanism whereby a RNA intermediate is reverse-transcribed into a cDNA copy that is integrated elsewhere in the genome [1]. For long terminal repeat (LTR) retrotransposons, integration occurs by means of a cleavage and strand-transfer reaction catalyzed by an integrase much like retroviruses [2]. For non-LTR retrotransposons, which include both long and short interspersed nuclear elements (LINEs and SINEs), chromosomal integration is coupled to the reverse transcription through a process referred to as target-primed reverse transcription [3]. Class 2 elements, also known as DNA transposons, are mobilized via a DNA intermediate, either directly through a ‘cut-and-paste’ mechanism [4, 5] or, in the case of *Helitrons*, a ‘peel-and-paste’ replicative mechanism involving a circular DNA intermediate [6]. For detailed reviews on individual TE types and transposition mechanisms, we refer the reader to the monograph edited by Craig et al. [7].

Each TE subclass is further divided into subgroups (or superfamilies) that are typically found across a wide range of organisms, but share a common genetic organization and a monophyletic origin. For example, Ty3/*gypsy* and Ty1/*copia* elements are two major superfamilies of LTR retrotransposons that occur in virtually all major groups of eukaryotes [8]. Similarly, Tc1/*mariner*, hAT (hobo-Ac-Tam3), and MULEs (Mutator-like elements) are three superfamilies of DNA transposons that are widespread across the eukaryotic tree [9]. At the most detailed level of TE classification, elements are grouped into families or subfamilies, which can be defined as a closely related group of elements that can be traced as descendants of a single ancestral unit [10]. This ancestral copy can be inferred as a consensus sequence, which is representative of the entire (sub)family [11, 12]. Thus, in principle, every TE sequence in a genome can be affiliated to a (sub)family, superfamily, subclass, and class (Fig. 1). However, much like the taxonomy of species, the classification of TEs is in constant flux, perpetually subject to revision due to the discovery of completely novel TE types, the introduction of new levels of granularity in the classification, and ongoing development of methods and criteria to detect and classify TEs [13, 14].

## TEs are not randomly distributed in the genome

**Fig. 2:**
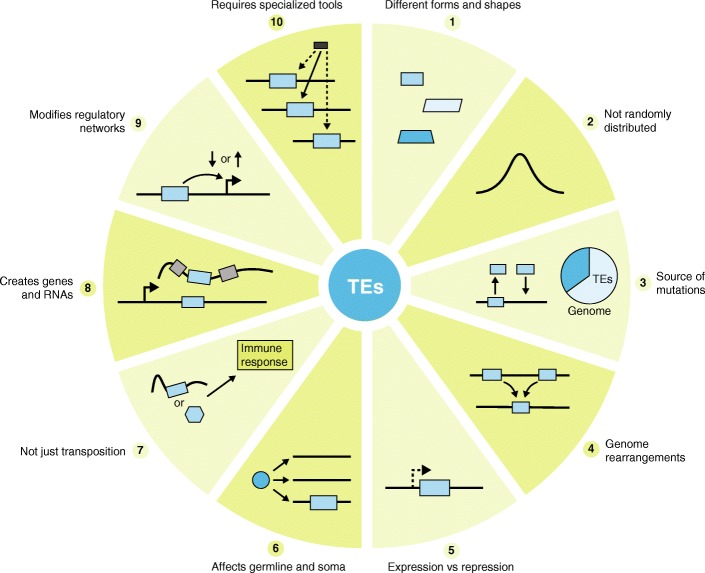
Ten things you should know about transposable elements (TEs). Examples of how TEs can impact genomes in direct and indirect ways. Blue boxes represent TEs, gray boxes represent canonical exons, and the black box represents a sequencing read. Right-angled arrows represent gene or TE promoters

The genome may be viewed as an ecosystem inhabited by diverse communities of TEs, which seek to propagate and multiply through sophisticated interactions with each other and with other components of the cell [15]. These interactions encompass processes familiar to ecologists, such as parasitism, cooperation, and competition [16]. Thus, it is perhaps not surprising that TEs are rarely, if ever, randomly distributed in the genome. TEs exhibit various levels of preference for insertion within certain features or compartments of the genome (Fig. 2). These are often guided by opposite selective forces, a balancing act of facilitating future propagation while mitigating deleterious effects on host cell function. At the most extreme end of the site-selection spectrum, many elements have evolved mechanisms to target specific loci where their insertions are less detrimental to the host but favorable for their propagation [17]. For instance, several retrotransposons in species as diverse as slime mold and budding and fission yeast have evolved independently, but convergently, the capacity to target the upstream regions of genes transcribed by RNA polymerase III, where they do not appear to affect host gene expression but retain the ability to be transcribed themselves [17–20].

Natural selection and genetic drift are also powerful forces shaping the distribution and accumulation of TEs [21]. Insertions that are strongly deleterious are rapidly removed from the population. Insertions that have little or no effects on genome function and host fitness may reach fixation according to the efficiency of selection and drift at purging these insertions from the population, which vary greatly among species [21]. Selective forces can explain why some elements are more likely to be retained in certain genomic locations than others [22, 23]. For instance, de novo insertions of the human LINE 1 (L1) retrotransposon readily occur within (and disrupt) gene exons [24], but very few if any L1 elements have been fixed within the coding region of human genes [25]. Similarly, no LTR retrotransposon is known to exhibit insertion preference with regard to which DNA strand is transcribed, and yet these elements are strongly depleted in the sense orientation within human introns—most likely due to their propensity to interfere with gene splicing and polyadenylation when inserted in sense orientation [11, 26]. Perhaps because of some of these shared properties, the evolutionary trajectories of TE accumulation in mammals were found to be conserved across species in spite of clade specific differences in TE content. [27]. Thus, the success and diversity of TEs in a genome are shaped both by properties intrinsic to the elements as well as evolutionary forces acting at the level of the host species. A solid comprehension of how these forces act together is paramount to understanding the impact of TEs on organismal biology.

## TEs are an extensive source of mutations and genetic polymorphisms

TEs occupy a substantial portion of the genome of a species, including a large fraction of the DNA unique to that species. In maize, where Barbara McClintock did her seminal work [28], an astonishing 60 to 70% of the genome is comprised of LTR retrotransposons, many of which are unique to this species or its close wild relatives, but the less prevalent DNA transposons are currently the most active and mutagenic [29–32] (Fig. 2). Similarly, the vast majority of TE insertions in *Drosophila melanogaster* are absent at the orthologous site in its closest relative *D. simulans* (and vice versa), and most are not fixed in the population [33, 34]. Many TE families are still actively transposing and the process is highly mutagenic; more than half of all known phenotypic mutants of *D. melanogaster* isolated in the laboratory are caused by spontaneous insertions of a wide variety of TEs [35]. Transposition events are also common and mutagenic in laboratory mice, where ongoing activity of several families of LTR elements are responsible for 10–15% of all inherited mutant phenotypes [36]. This contribution of TEs to genetic diversity may be underestimated, as TEs can be more active when organisms are under stress, such as in their natural environment [37, 38].

Because TE insertions rarely provide an immediate fitness advantage to their host, those reaching fixation in the population do so largely by genetic drift and are subsequently eroded by point mutations that accumulate neutrally [21]. Over time, these mutations result in TEs that can no longer encode transposition enzymes and produce new integration events. For instance, our (haploid) genome contains ~ 500,000 L1 copies, but more than 99.9% of these L1 copies are fixed and no longer mobile due to various forms of mutations and truncations [39, 40]. It is estimated that each person carries a set of ~ 100 active L1 elements, and most of these are young insertions still segregating within the human population [41–43]. Thus, as for any other organism, the ‘reference’ human genome sequence does not represent a comprehensive inventory of TEs in humans. Thousands of ‘non-reference’, unfixed TE insertions have been catalogued through whole genome sequencing and other targeted approaches [44]. On average, any two human haploid genomes differ by approximately a thousand TE insertions, primarily from the L1 or Alu families. The number of TE insertion polymorphisms in a species with much higher TE activity such as maize [32] dwarfs the number in humans.

If TEs bring no immediate benefit to their host and are largely decaying neutrally once inserted, how do they persist in evolution? One key to this conundrum is the ability of TEs not only to propagate vertically but also horizontally between individuals and species. There is now a large body of evidence supporting the idea that horizontal transposon transfer is a common phenomenon that affects virtually every major type of TE and all branches of the tree of life [45, 46]. While the cellular mechanisms underlying horizontal transposon transfer remain murky, it is increasingly apparent that the intrinsic mobility of TEs and ecological interactions between their host species, including those with pathogens and parasites, facilitate the transmission of elements between widely diverged taxa [47–49].

## TEs are associated with genome rearrangements and unique chromosome features

Transposition represents a potent mechanism of genome expansion that over time is counteracted by the removal of DNA via deletion. The balance between the two processes is a major driver in the evolution of genome size in eukaryotes [21, 50, 51]. Several studies have demonstrated the impact and range of this shuffling and cycling of genomic content on the evolution of plant and animal genomes [52–55]. Because the insertion and removal of TEs is often imprecise, these processes can indirectly affect surrounding host sequences. Some of these events occur at high enough frequency to result in vast amounts of duplication and reshuffling of host sequences, including genes and regulatory sequences. For example, a single group of DNA transposons (MULEs) has been responsible for the capture and reshuffling of ~ 1,000 gene fragments in the rice genome [56]. Such studies have led to the conclusion that the rate at which TEs transpose, which is in part under host control, is an important driver of genome evolution [57–59].

In addition to rearrangements induced as a byproduct of transposition, TEs can promote genomic structural variation long after they have lost the capacity to mobilize [60]. In particular, recombination events can occur between the highly homologous regions dispersed by related TEs at distant genomic positions and result in large-scale deletions, duplications, and inversions [59, 61–63] (Fig. 2). TEs also provide regions of microhomology that predispose to template switching during repair of replication errors leading to another source of structural variants [64]. These non-transposition-based mechanisms for TE-induced or TE-enabled structural variation have contributed substantially to genome evolution. These processes can also make the identification of actively transposing elements more difficult in population studies that infer the existence of active elements through the detection of non-reference insertions.

TEs also contribute to specialized chromosome features. An intriguing example is in *Drosophila*, where LINE-like retrotransposons form and maintain the telomeres in replacement of the telomerase enzyme which has been lost during dipteran evolution [65]. This domestication event could be viewed as a replay of what might have happened much earlier in eukaryotic evolution to solve the ‘end problem’ created by the linearization of chromosomes. Indeed, the reverse transcriptase component of telomerase is thought to have originated from an ancient lineage of retroelements [66, 67]. TE sequences and domesticated transposase genes also play structural roles at centromeres [68–70].

## There is an intrinsic balance between TE expression and repression

To persist in evolution, TEs must strike a delicate balance between expression and repression (Fig. 2). Expression should be sufficient to promote amplification, but not so vigorous as to lead to a fitness disadvantage for the host that would offset the benefit to the TE of increased copy numbers. This balancing act may explain why TE-encoded enzymes are naturally suboptimal for transposition [71, 72] and why some TEs have evolved self-regulatory mechanisms controlling their own copy numbers [73, 74]. A variety of host factors are also employed to control TE expression, which includes a variety of small RNA, chromatin, and DNA modification pathways [75–78], as well as sequence-specific repressors such as the recently profiled KRAB zinc-finger proteins [79–82]. However, many of these silencing mechanisms must be at least partially released to permit developmental regulation of host gene expression programs, particularly during early embryonic development. For example, genome-wide loss of DNA methylation is necessary to reset imprinted genes in primordial germ cells [83]. This affords TEs an opportunity, as reduced DNA methylation often promotes TE expression. Robust expression of a TE in the germ lineage (but not necessarily in the gametes themselves) is often its own downfall. In one example of a clever trick employed by the host, TE repression is relieved in a companion cell derived from the same meiotic product as flowering plant sperm [84]. However, this companion cell does not contribute genetic material to the next generation. Thus, although TEs transpose in a meiotic product, the events are not inherited. Instead, TE activity in the companion cell may further dampen TE activity in sperm via the import of TE-derived small RNAs [85].

Another important consequence of the intrinsic expression/repression balance is that the effects of TEs on a host can vary considerably among tissue types and stages of an organism’s life cycle. From the TE’s perspective, an ideal scenario is to be expressed and active in the germline, but not in the soma, where expression would gain the TE no advantage, only disadvantage [86]. This is indeed observed among many species, with ciliates representing an extreme example of this division—TEs are actively deleted from the somatic macronucleus but retained in the micronucleus, or germline [87]. Another example is the P-elements in *Drosophila*, which are differentially spliced in the germline versus soma [88]. Many organisms, including plants, do not differentiate germ lineage cells early in development; rather, they are specified from somatic cells shortly before meiosis commences. Thus, TEs that transpose in somatic cells in plants have the potential to be inherited, which suggests that the interest of TEs and host are in conflict across many more cells and tissues than in animals with a segregated germline.

## TEs are insertional mutagens in both germline and soma

Like other species, humans contend with a contingent of currently active TEs where the intrinsic balance between expression and repression is still at play [89]. For us, this includes L1 and other mobile elements that depend on L1-encoded proteins for retrotransposition [90, 91]. These elements are responsible for new germline insertions that can cause genetic disease. More than 120 independent TE insertions have been associated with human disease [24]. The rate of de novo germline transposition in humans is approximately one in 21 births for *Alu* [92] and one in 95 births for L1 [93].

Historically, little attention has been given to transposition in somatic cells and its consequences, because somatic transposition may be viewed as an evolutionary dead-end for the TE with no long-term consequences for the host species. Yet, there is abundant evidence that TEs are active in somatic cells in many organisms [94] (Fig. 2). In humans, L1 expression and transposition have been detected in a variety of somatic contexts, including early embryos and certain stem cells [95, 96]. There is also a great deal of interest in mobile element expression and activity in the mammalian brain, where L1 transposition has been proposed to diversify neuronal cell populations [97–99]. One challenge for assessing somatic activity has rested with the development of reliable single cell insertion site mapping strategies [100–103].

Somatic activity has also been observed in human cancers, where tumors can acquire hundreds of new L1 insertions [104–109]. Just like for human polymorphisms, somatic activity in human cancers is caused by small numbers of so-called ‘hot’ L1 loci [41, 107]. The activities of these master copies varies depending on the individual [105], tumor type [105], and timeframe in the clonal evolution of the tumor [106, 110]. Some of these de novo L1 insertions disrupt critical tumor suppressors and oncogenes and thus drive cancer formation [107], although the vast majority appear to be ‘passenger’ mutations [111]. Host cells have evolved several mechanisms to keep TEs in check. However, as the force of natural selection begins to diminish with age and completely drops in post-reproductive life, TEs may become more active [112].

## TEs can be damaging in ways that do not involve transposition

TEs are best known for their mobility, in other words their ability to transpose to new locations. While the breakage and insertion of DNA associated with transposition represents an obvious source of cell damage, this is not the only or perhaps even the most common mechanism by which TEs can be harmful to their host. Reactivated transposons harm the host in multiple ways. First, de-repression of transposon loci, including their own transcription, may interfere with transcription or processing of host mRNAs through a myriad of mechanisms [113–115]. Genome-wide transcriptional de-repression of TEs has been documented during replicative senescence of human cells [116] and several mouse tissues, including liver, muscle, and brain [117, 118]. De-repression of LTR and L1 promoters can also cause oncogene activation in cancer [119]. Second, TE-encoded proteins such as the endonuclease activity of L1 ORF2p can induce DNA breaks and genomic instability [120]. Third, accumulation of RNA transcripts and extrachromosomal DNA copies derived from TEs may trigger an innate immune response leading to autoimmune diseases and sterile inflammation (Fig. 2). Activation of interferon response is now a well-documented property of transcripts derived from endogenous retroviruses and may give immunotherapies a boost in identifying and attacking cancer cells [121–123]. The relative contribution of all the above mechanisms in organismal pathologies remains to be determined.

Following transcription (and sometimes splicing) of TEs, the next step in the process involves translation of the encoded proteins and, for retroelements, reverse transcription of the TEs into cDNA substrates suitable for transposition. Once engaged by a TE-encoded reverse transcriptase protein, the resulting cytosolic DNAs and RNA:DNA hybrids can alert inflammatory pathways. An example of this is seen in patients with Aicardi–Goutières syndrome, where accumulation of TE-derived cytosolic DNA is due to mutations in pathways that normally block TE processing or degrade TE-derived DNA [124, 125]. Although not all TEs encode functional proteins, some do, including a few endogenous retroviruses capable of producing Gag, Pol, or envelope (Env) proteins [126]. Overexpression of these Env proteins can be cytotoxic, and has been linked to at least two neurodegenerative diseases, multiple sclerosis [127] and amytrophic lateral sclerosis [128]. Small accessory proteins produced by the youngest human endogenous retrovirus (HERV) group, HERV-K (HML-2), may play a role in some cancers but the evidence remains circumstantial [129, 130].

## A number of key coding and non-coding RNAs are derived from TEs

Although usually detrimental, there is growing evidence that TE insertions can provide raw material for the emergence of protein-coding genes and non-coding RNAs, which can take on important and, in some cases essential, cellular function [131–133] (Fig. 2). The process of TE gene ‘domestication’ or exaptation over evolutionary time contributes to both deeply conserved functions and more recent, species-specific traits. Most often, the ancestral or a somewhat modified role of a TE-encoded gene is harnessed by the host and conserved, while the rest of the TE sequence, and hence its ability to autonomously transpose, has been lost. Spectacular examples of deeply conserved TE-derived genes are *Rag1* and *Rag2*, that catalyze V(D)J somatic recombination in the vertebrate immune system. Both genes, and probably the DNA signals they recognize, were derived from an ancestral DNA transposon around 500 million years ago [134, 135]. Indeed, DNA transposases have been co-opted multiple times to form new cellular genes [70, 113].

The *gag* and *env* genes of LTR retrotransposons or endogenous retroviruses (ERVs) have also been domesticated numerous times to perform functions in placental development, contribute to host defense against exogenous retroviruses, act in brain development, and play other diverse roles [132, 136]. One of the most intriguing examples of TE domestication is the repeated, independent capture of ERV *env* genes, termed *syncytins*, which appear to function in placentation by facilitating cell–cell fusion and syncytiotrophoblast formation [137–139]. Notably, one or more such *syncytin* genes have been found in virtually every placental mammalian lineage where they have been sought, strongly suggesting that ERVs have played essential roles in the evolution and extreme phenotypic variability of the mammalian placenta. Another example of a viral-like activity re-purposed for host cell function is provided by the neuronal *Arc* gene, which arose from the *gag* gene from a LTR retrotransposon domesticated in the common ancestor of tetrapod vertebrates [140]. Genetic and biochemical studies of murine Arc show that it is involved in memory and synaptic plasticity and has preserved most of the ancestral activities of Gag, including the packaging and intercellular trafficking of its own RNA [140]. Remarkably, flies appear to have independently evolved a similar system of trans-synaptic RNA delivery involving a *gag*-like protein derived from a similar yet distinct lineage of LTR retrotransposons [141]. Thus, the biochemical activities of TE-derived proteins have been repeatedly co-opted during evolution to foster the emergence of convergent cellular innovations in different organisms.

TEs can donate their own genes to the host, but they can also add exons and rearrange and duplicate existing host genes. In humans, intronic *Alu* elements are particularly prone to be captured as alternative exons through cryptic splice sites residing within their sequences [142, 143]. L1 and SVA (SINE/VNTR/Alu) elements also contribute to exon shuffling through transduction events of adjacent host sequences during their mobilization [144, 145]. The reverse transcriptase activity of retroelements is also responsible for the trans-duplication of cellular mRNAs to create ‘processed’ retrogenes in a wide range of organisms [146, 147]. The L1 enzymatic machinery is thought to be involved in the generation of tens of thousands of retrogene copies in mammalian genomes, many of which remain transcribed and some of which have acquired new cellular functions [147, 148]. This is a process still actively shaping our genomes; it has been estimated that 1 in every 6000 humans carries a novel retrogene insertion [93].

TEs also make substantial contributions to non-protein coding functions of the cell. They are major components of thousands of long non-coding RNAs in human and mouse genomes, often transcriptionally driven by retroviral LTRs [149]. Some of these TE-driven lncRNAs appear to play important roles in the maintenance of stem cell pluripotency and other developmental processes [150–154]. Many studies have demonstrated that TE sequences embedded within lncRNAs and mRNAs can directly modulate RNA stability, processing, or localization with important regulatory consequences [114, 155–158]. Furthermore, TE-derived microRNAs [159] and other small RNAs processed from TEs [160] can also adopt regulatory roles serving host cell functions. The myriad of mechanisms by which TEs contribute to coding and non-coding RNAs illustrate the multi-faceted interactions between these elements and their host.

## TEs contribute cis-regulatory DNA elements and modify transcriptional networks

Cis-regulatory networks coordinate the transcription of multiple genes that function in concert to orchestrate entire pathways and complex biological processes. In line with Barbara McClintock’s insightful predictions [28], there is now mounting evidence that TEs have been a rich source of material for the modulation of eukaryotic gene expression (Fig. 2). Indeed, TEs can disperse vast amounts of promoters and enhancers [161–166], transcription factor binding sites [167–172], insulator sequences [173–175], and repressive elements [176, 177] (reviewed in [178]). The varying coat colors of agouti mice provides a striking example of a host gene controlling coat color whose expression can be altered by the methylation levels of a TE upstream of its promoter [179, 180]. In the oil palm, the methylation level of a TE that sits within a gene important for flowering ultimately controls whether or not the plants bear oil-rich fruit [181].

As TE families typically populate a genome as a multitude of related copies, it has long been postulated that they have the potential to donate the same cis-regulatory module to ‘wire’ batteries of genes dispersed throughout the genome [182]. An increasing number of studies support this model and suggest that TEs have provided the building blocks for the assembly and remodeling of cis-regulatory networks during evolution, including pathways underlying processes as diverse as pregnancy [183, 184], stem cell pluripotency [150, 151, 171], neocortex development [185], innate immunity in mammals [163], or the response to abiotic stress in maize [186]. Indeed, TE sequences harbor all the necessary features of a ‘classical’ gene regulatory network [113, 114]. They are bound by diverse sets of transcription factors [172] integrate multiple inputs (activation/repression), respond to signals in both *cis* and *trans*, and are capable of co-ordinately regulating gene expression. In this context, TEs are highly suitable agents to modify biological processes by creating novel cis-regulatory circuits and fine-tuning pre-existing networks.

## Analyzing TEs requires specialized tools

TEs have been historically neglected and remain frequently ignored in genomic studies in part because of their repetitive nature, which poses a number of analytical challenges and often requires the use of specialized tools [187]. As genomes can harbor thousands of copies of very similar TE sequences, uniqueness or, alternatively, repetitiveness of substrings within these regions need to be taken into consideration during both experimental design and analysis. As an example, short DNA oligos targeting a specific TE instance in the genome for PCR, short hairpin RNA, or CRISPR-Cas9 have to be carefully designed and validated to ensure that they are truly specific and target unique regions of the genome. In some scenarios, it can be acceptable or even desirable to target many elements simultaneously [150] or an entire TE family [153, 188–191].

Similarly, uniqueness and repetitiveness are important concepts to consider when aligning reads from next generation sequencing and analyzing TEs (Fig. 2). Various strategies exist to assign reads that could originate from multiple genomic locations: 1) mapping reads to consensus sequences of TE subfamilies [172]; 2) mapping to the genome and keeping only uniquely-mapping reads [163, 168]; 3) assigning multiple mapping reads at random between possible candidates [192]; or 4) redistributing them according to various algorithms, such as maximum likelihood [193, 194]. The choice is ultimately guided by the technique (such as ChIP-seq and RNA-seq) and the purpose of the analysis—is information about individual TE instances needed, or is a high-level tally of results for each subfamily sufficient? Notably, these issues of uniqueness will differ substantially depending on the species studied and the presence or absence of recently, or currently, active TE families. For example, mapping reads to TEs in the human genome will be less challenging than in the mouse genome given the more recent and mobile TE landscape of the latter species [36]. Finally, as sequencing technology and bioinformatics pipelines improve, notably with the increasing length of sequencing reads, many of the hurdles faced by earlier studies will be progressively removed [187].

## Outlook

As potent insertional mutagens, TEs can have both positive and negative effects on host fitness, but it is likely that the majority of TE copies in any given species—and especially those such as humans with small effective population size—have reached fixation through genetic drift alone and are now largely neutral to their host. When can we say that TEs have been co-opted for cellular function? The publication of the initial ENCODE paper [195], which asserted ‘function for 80% of the genome’, was the subject of much debate and controversy. Technically speaking, ENCODE assigned only ‘biochemical’ activity to this large fraction of the genome. Yet critics objected to the grand proclamations in the popular press (The Washington Post Headline: “Junk DNA concept debunked by new analysis of the human genome”) and to the ENCODE consortium’s failure to prevent this misinterpretation [196–198]. To these critics, ignoring evolutionary definitions of function was a major misstep.

This debate can be easily extended to include TEs. TEs make up the vast majority of what is often referred to as ‘junk DNA’. Today, the term is mostly used (and abused) by the media, but it has in fact deep roots in evolutionary biology [199]. Regardless of the semantics, what evidence is needed to assign a TE with a function? Many TEs encode a wide range of biochemical activities that normally benefit their own propagation. For example, TEs often contain promoter or enhancer elements that highjack cellular RNA polymerases for transcription and autonomous elements encode proteins with various biochemical and enzymatic activities, all of which are necessary for the transposon to replicate. Do these activities make them functional?

The vast differences in TEs between species make standard approaches to establish their regulatory roles particularly challenging [200]. For example, intriguing studies on the impact of HERVs, in particular HERV-H, in stem cells and pluripotency [150–152] must be interpreted using novel paradigms that do not invoke deep evolutionary conservation to imply function, as these particular ERVs are absent outside of great apes. Evolutionary constraint can be measured at shorter time scales, including the population level, but this remains a statistically challenging task especially for non-coding sequences. Natural loss-of-function alleles may exist in the human population and their effect on fitness can be studied if their impact is apparent, but these are quite rare and do not allow systematic studies. It is possible to engineer genetic knockouts of a particular human TE locus to test its regulatory role but those are restricted to in-vitro systems, especially when the orthologous TE does not exist in the model species. In this context, studying the impact of TEs in model species with powerful genome engineering tools and vast collections of mutants and other genetic resources, such as plants, fungi, and insects, will also continue to be extremely valuable.

Finally, a growing consensus is urging more rigor when assigning cellular function to TEs, particularly for the fitness benefit of the host [178]. Indeed, a TE displaying biochemical activity (such as those bound by transcription factors or lying within open chromatin regions) cannot be equated to a TE that shows evidence of purifying selection at the sequence level or, when genetically altered, result in a deleterious or dysfunctional phenotype. Recent advances in editing and manipulating the genome and the epigenome en masse yet with precision, including repetitive elements [153, 154, 189–191], offer the promise for a systematic assessment of the functional significance of TEs.

## Abbreviations

Env: Envelope protein
ERV: Endogenous retrovirus
HERV: Human endogenous retrovirus
L1: Long interspersed nuclear element 1
LINE: Long interspersed nuclear element
LTR: Long terminal repeat
SINE: Short interspersed nuclear element
TE: Transposable element

## References

[1] Boeke JD, Garfinkel DJ, Styles CA, Fink GR (1985). Ty elements transpose through an RNA intermediate. Cell.

[2] Brown PO, Bowerman B, Varmus HE, Bishop JM (1987). Correct integration of retroviral DNA in vitro. Cell.

[3] Luan DD, Korman MH, Jakubczak JL, Eickbush TH (1993). Reverse transcription of R2Bm RNA is primed by a nick at the chromosomal target site: a mechanism for non-LTR retrotransposition. Cell.

[4] Greenblatt IM, Brink RA (1963). Transpositions of modulator in maize into divided and undivided chromosome segments. Nature.

[5] Rubin GM, Kidwell MG, Bingham PM (1982). The molecular basis of P-M hybrid dysgenesis: the nature of induced mutations. Cell.

[6] Grabundzija I, Messing SA, Thomas J, Cosby RL, Bilic I, Miskey C (2016). A Helitron transposon reconstructed from bats reveals a novel mechanism of genome shuffling in eukaryotes. Nat Commun.

[7] Craig NL, Chandler M, Gellert M, Lambowitz AM, Rice PA, Sandmeyer SB. Mobile DNA III. 3rd ed. Washington, DC: American Society for Microbiology (ASM); 2015.

[8] Malik HS, Eickbush TH (2001). Phylogenetic analysis of ribonuclease H domains suggests a late, chimeric origin of LTR retrotransposable elements and retroviruses. Genome Res.

[9] Feschotte C, Pritham EJ (2007). DNA transposons and the evolution of eukaryotic genomes. Annu Rev Genet.

[10] Britten RJ, Kohne DE (1968). Repeated sequences in DNA. Science.

[11] Smit AF (1999). Interspersed repeats and other mementos of transposable elements in mammalian genomes. Curr Opin Genet Dev.

[12] Jurka J, Smith T (1988). A fundamental division in the Alu family of repeated sequences. Proc Natl Acad Sci U S A.

[13] Wicker T, Sabot F, Hua-Van A, Bennetzen JL, Capy P, Chalhoub B (2007). A unified classification system for eukaryotic transposable elements. Nat Rev Genet.

[14] Arkhipova IR (2017). Using bioinformatic and phylogenetic approaches to classify transposable elements and understand their complex evolutionary histories. Mob DNA.

[15] Venner S, Feschotte C, Biémont C (2009). Dynamics of transposable elements: towards a community ecology of the genome. Trends Genet.

[16] Robillard É, Rouzic AL, Zhang Z, Capy P, Hua-Van A (2016). Experimental evolution reveals hyperparasitic interactions among transposable elements. Proc Natl Acad Sci U S A.

[17] Sultana T, Zamborlini A, Cristofari G, Lesage P (2017). Integration site selection by retroviruses and transposable elements in eukaryotes. Nat Rev Genet.

[18] Spaller T, Kling E, Glöckner G, Hillmann F, Winckler T (2016). Convergent evolution of tRNA gene targeting preferences in compact genomes. Mob DNA.

[19] Cheung S, Manhas S, Measday V (2018). Retrotransposon targeting to RNA polymerase III-transcribed genes. Mob DNA.

[20] Guo Y, Singh PK, Levin HL (2015). A long terminal repeat retrotransposon of *Schizosaccharomyces japonicus* integrates upstream of RNA pol III transcribed genes. Mob DNA.

[21] Lynch M (2007). The origins of genome architecture.

[22] Campos-Sánchez R, Cremona MA, Pini A, Chiaromonte F, Makova KD (2016). Integration and fixation preferences of human and mouse endogenous retroviruses uncovered with functional data analysis. PLOS Comput Biol.

[23] Kvikstad EM, Makova KD (2010). The (r)evolution of SINE versus LINE distributions in primate genomes: sex chromosomes are important. Genome Res.

[24] Hancks DC, Kazazian HH (2016). Roles for retrotransposon insertions in human disease. Mob DNA.

[25] Gotea V, Makalowski W (2006). Do transposable elements really contribute to proteomes?. Trends Genet.

[26] Medstrand P, Van De Lagemaat LN, Mager DL (2002). Retroelement distributions in the human genome: variations associated with age and proximity to genes. Genome Res.

[27] Buckley RM, Kortschak RD, Raison JM, Adelson DL (2017). Similar evolutionary trajectories for retrotransposon accumulation in mammals. Genome Biol Evol.

[28] McClintock B. (1956). Controlling Elements and the Gene. Cold Spring Harbor Symposia on Quantitative Biology.

[29] Schnable PS, Ware D, Fulton RS, Stein JC, Wei F, Pasternak S (2009). The B73 maize genome: complexity, diversity, and dynamics. Science.

[30] Lazarow K, Doll M-L, Kunze R, Peterson T (2013). Molecular Biology of Maize Ac/Ds elements: an overview. Plant transposable elements.

[31] Liu S, Yeh C-T, Ji T, Ying K, Wu H, Tang HM (2009). Mu transposon insertion sites and meiotic recombination events co-localize with epigenetic marks for open chromatin across the maize genome. PLoS Genet.

[32] Springer NM, Anderson SN, Andorf CM, Ahern KR, Bai F, Barad O (2018). The maize W22 genome provides a foundation for functional genomics and transposon biology. Nat Genet.

[33] Kofler R, Nolte V, Schlötterer C (2015). Tempo and mode of transposable element activity in Drosophila. PLOS Genet.

[34] Rahman R, Chirn G, Kanodia A, Sytnikova YA, Brembs B, Bergman CM (2015). Unique transposon landscapes are pervasive across *Drosophila melanogaster* genomes. Nucleic Acids Res.

[35] Eickbush TH, Furano AV (2002). Fruit flies and humans respond differently to retrotransposons. Curr Opin Genet Dev.

[36] Maksakova IA, Romanish MT, Gagnier L, Dunn CA, Van de Lagemaat LN, Mager DL (2006). Retroviral elements and their hosts: insertional mutagenesis in the mouse germ line. PLoS Genet.

[37] Lanciano S, Mirouze M (2018). Transposable elements: all mobile, all different, some stress responsive, some adaptive?. Curr Opin Genet Dev.

[38] Horváth V, Merenciano M, González J (2017). Revisiting the relationship between transposable elements and the eukaryotic stress response. Trends Genet.

[39] Ostertag EM, Kazazian HH (2001). Biology of mammalian L1 retrotransposons. Annu Rev Genet.

[40] Szak ST, Pickeral OK, Makalowski W, Boguski MS, Landsman D, Boeke JD (2002). Molecular archeology of L1 insertions in the human genome. Genome Biol.

[41] Brouha B, Schustak J, Badge RM, Lutz-Prigge S, Farley AH, Moran JV (2003). Hot L1s account for the bulk of retrotransposition in the human population. Proc Natl Acad Sci U S A.

[42] Sassaman DM, Dombroski BA, Moran JV, Kimberland ML, Naas TP, DeBerardinis RJ (1997). Many human L1 elements are capable of retrotransposition. Nat Genet.

[43] Beck CR, Collier P, Macfarlane C, Malig M, Kidd JM, Eichler EE (2010). LINE-1 retrotransposition activity in human genomes. Cell.

[44] Sudmant PH, Rausch T, Gardner EJ, Handsaker RE, Abyzov A, Huddleston J (2015). An integrated map of structural variation in 2,504 human genomes. Nature.

[45] Gilbert C, Feschotte C (2018). Horizontal acquisition of transposable elements and viral sequences: patterns and consequences. Curr Opin Genet Dev.

[46] Wallau GL, Vieira C, Loreto ÉLS (2018). Genetic exchange in eukaryotes through horizontal transfer: connected by the mobilome. Mob DNA.

[47] Gilbert C, Cordaux R (2017). Viruses as vectors of horizontal transfer of genetic material in eukaryotes. Curr Opin Virol.

[48] Metzger MJ, Paynter AN, Siddall ME, Goff SP (2018). Horizontal transfer of retrotransposons between bivalves and other aquatic species of multiple phyla. Proc Natl Acad Sci U S A.

[49] Ivancevic AM, Kortschak RD, Bertozzi T, Adelson DL (2018). Horizontal transfer of BovB and L1 retrotransposons in eukaryotes. Genome Biol.

[50] Petrov DA (2002). Mutational equilibrium model of genome size evolution. Theor Popul Biol.

[51] Schubert I, Vu GTH (2016). Genome stability and evolution: attempting a holistic view. Trends Plant Sci.

[52] Gregory TR, Johnston JS (2008). Genome size diversity in the family Drosophilidae. Heredity.

[53] Kapusta A, Suh A, Feschotte C (2017). Dynamics of genome size evolution in birds and mammals. Proc Natl Acad Sci U S A.

[54] Pellicer J, Kelly LJ, Leitch IJ, Zomlefer WB, Fay MF (2014). A universe of dwarfs and giants: genome size and chromosome evolution in the monocot family Melanthiaceae. New Phytol.

[55] Thybert D, Roller M, Navarro FCP, Fiddes I, Streeter I, Feig C (2018). Repeat associated mechanisms of genome evolution and function revealed by the Mus caroli and Mus pahari genomes. Genome Res.

[56] Jiang N, Bao Z, Zhang X, Eddy SR, Wessler SR (2004). Pack-MULE transposable elements mediate gene evolution in plants. Nature.

[57] Cordaux R, Batzer MA (2009). The impact of retrotransposons on human genome evolution. Nat Rev Genet.

[58] Freeling M, Xu J, Woodhouse M, Lisch D (2015). A solution to the C-value paradox and the function of junk DNA: the genome balance hypothesis. Mol Plant.

[59] Bennetzen JL, Wang H (2014). The contributions of transposable elements to the structure, function, and evolution of plant genomes. Annu Rev Plant Biol.

[60] Carvalho CM, Lupski JR (2016). Mechanisms underlying structural variant formation in genomic disorders. Nat Rev Genet.

[61] Deininger PL, Moran JV, Batzer MA, Kazazian HH (2003). Mobile elements and mammalian genome evolution. Curr Opin Genet Dev.

[62] Ade C, Roy-Engel AM, Deininger PL (2013). Alu elements: an intrinsic source of human genome instability. Curr Opin Virol.

[63] Han K, Lee J, Meyer TJ, Remedios P, Goodwin L, Batzer MA (2008). L1 recombination-associated deletions generate human genomic variation. Proc Natl Acad Sci U S A.

[64] Lee JA, Carvalho CM, Lupski JR (2007). A DNA replication mechanism for generating nonrecurrent rearrangements associated with genomic disorders. Cell.

[65] Pardue M-L, DeBaryshe PG (2011). Retrotransposons that maintain chromosome ends. Proc Natl Acad Sci U S A.

[66] Belfort M, Curcio MJ, Lue NF (2011). Telomerase and retrotransposons: reverse transcriptases that shaped genomes. Proc Natl Acad Sci U S A.

[67] Fulcher N, Derboven E, Valuchova S, Riha K (2014). If the cap fits, wear it: an overview of telomeric structures over evolution. Cell Mol Life Sci.

[68] Casola C, Hucks D, Feschotte C (2007). Convergent domestication of pogo-like transposases into centromere-binding proteins in fission yeast and mammals. Mol Biol Evol.

[69] Kursel LE, Malik HS (2016). Centromeres. Curr Biol.

[70] Jangam D, Feschotte C, Betrán E (2017). Transposable element domestication as an adaptation to evolutionary conflicts. Trends Genet.

[71] Lampe DJ, Akerley BJ, Rubin EJ, Mekalanos JJ, Robertson HM (1999). Hyperactive transposase mutants of the Himar1 mariner transposon. Proc Natl Acad Sci U S A.

[72] Mátés L, Chuah MKL, Belay E, Jerchow B, Manoj N, Acosta-Sanchez A (2009). Molecular evolution of a novel hyperactive *Sleeping Beauty* transposase enables robust stable gene transfer in vertebrates. Nat Genet.

[73] Lohe AR, Hartl DL (1996). Autoregulation of mariner transposase activity by overproduction and dominant-negative complementation. Mol Biol Evol.

[74] Saha A, Mitchell JA, Nishida Y, Hildreth JE, Ariberre JA, Gilbert WV (2015). A trans-dominant form of gag restricts Ty1 retrotransposition and mediates copy number control. J Virol.

[75] Molaro A, Malik HS (2016). Hide and seek: how chromatin-based pathways silence retroelements in the mammalian germline. Curr Opin Genet Dev.

[76] Liu N, Lee CH, Swigut T, Grow E, Gu B, Bassik MC (2017). Selective silencing of euchromatic L1s revealed by genome-wide screens for L1 regulators. Nature.

[77] Goodier JL (2016). Restricting retrotransposons: a review. Mob DNA.

[78] Berrens RV, Andrews S, Spensberger D, Santos F, Dean W, Gould P (2017). An endosiRNA-based repression mechanism counteracts transposon activation during global dna demethylation in embryonic stem cells. Cell Stem Cell.

[79] Imbeault M, Trono D (2014). As time goes by: KRABs evolve to KAP endogenous retroelements. Dev Cell.

[80] Imbeault M, Helleboid P-Y, Trono D (2017). KRAB zinc-finger proteins contribute to the evolution of gene regulatory networks. Nature.

[81] Yang P, Wang Y, Macfarlan TS (2017). The role of KRAB-ZFPs in transposable element repression and mammalian evolution. Trends Genet.

[82] Ecco G, Imbeault M, Trono D (2017). KRAB zinc finger proteins. Development.

[83] Miyoshi N, Stel JM, Shioda K, Qu N, Odahima J, Mitsunaga S (2016). Erasure of DNA methylation, genomic imprints, and epimutations in a primordial germ-cell model derived from mouse pluripotent stem cells. Proc Natl Acad Sci U S A.

[84] Slotkin RK, Vaughn M, Borges F, Tanurdžić M, Becker JD, Feijó JA (2009). Epigenetic reprogramming and small RNA silencing of transposable elements in pollen. Cell.

[85] Martínez G, Panda K, Köhler C, Slotkin RK (2016). Silencing in sperm cells is directed by RNA movement from the surrounding nurse cell. Nat Plants.

[86] Haig D (2016). Transposable elements: self-seekers of the germline, team-players of the soma. BioEssays.

[87] Allen SE, Nowacki M (2017). Necessity is the mother of invention: ciliates, transposons, and transgenerational inheritance. Trends Genet.

[88] Teixeira FK, Okuniewska M, Malone CD, Coux R-X, Rio DC, Lehmann R (2017). piRNA-mediated regulation of transposon alternative splicing in the soma and germ line. Nature.

[89] Huang CRL, Burns KH, Boeke JD (2012). Active transposition in genomes. Annu Rev Genet.

[90] Beck CR, Garcia-Perez JL, Badge RM, Moran JV (2011). LINE-1 elements in structural variation and disease. Annu Rev Genomics Hum Genet.

[91] Burns KH, Boeke JD (2012). Human transposon tectonics. Cell.

[92] Xing J, Zhang Y, Han K, Salem AH, Sen SK, Huff CD (2009). Mobile elements create structural variation analysis of a complete human genome. Genome Res.

[93] Ewing AD, Kazazian HH (2010). High-throughput sequencing reveals extensive variation in human-specific L1 content in individual human genomes. Genome Res.

[94] Kazazian HH (2011). Mobile DNA transposition in somatic cells. BMC Biol.

[95] Garcia-Perez JL, Marchetto MC, Muotri AR, Coufal NG, Gage FH, O’shea KS (2007). LINE-1 retrotransposition in human embryonic stem cells. Hum Mol Genet.

[96] Klawitter S, Fuchs NV, Upton KR, Munoz-Lopez M, Shukla R, Wang J (2016). Reprogramming triggers endogenous L1 and Alu retrotransposition in human induced pluripotent stem cells. Nat Commun.

[97] Baillie JK, Barnett MW, Upton KR, Gerhardt DJ, Richmond TA, De Sapio F (2011). Somatic retrotransposition alters the genetic landscape of the human brain. Nature.

[98] Erwin JA, Marchetto MC, Gage FH (2014). Mobile DNA elements in the generation of diversity and complexity in the brain. Nat Rev Neurosci.

[99] Muotri AR, Chu VT, Marchetto MC, Deng W, Moran JV, Gage FH (2005). Somatic mosaicism in neuronal precursor cells mediated by L1 retrotransposition. Nature.

[100] Evrony GD, Lee E, Park PJ, Walsh CA (2016). Resolving rates of mutation in the brain using single-neuron genomics. Elife.

[101] Upton KR, Gerhardt DJ, Jesuadian JS, Richardson SR, Sánchez-Luque FJ, Bodea GO (2015). Ubiquitous L1 mosaicism in hippocampal neurons. Cell.

[102] Treiber CD, Waddell S (2017). Resolving the prevalence of somatic transposition in *Drosophila*. eLife.

[103] Faulkner GJ, Garcia-Perez JL (2017). L1 mosaicism in mammals: extent, effects, and evolution. Trends Genet.

[104] Iskow RC, McCabe MT, Mills RE, Torene S, Pittard WS, Neuwald AF (2010). Natural mutagenesis of human genomes by endogenous retrotransposons. Cell.

[105] Lee E, Iskow R, Yang L, Gokcumen O, Haseley P, Luquette LJ (2012). Landscape of somatic retrotransposition in human cancers. Science.

[106] Tubio JM, Li Y, Ju YS, Martincorena I, Cooke SL, Tojo M (2014). Extensive transduction of nonrepetitive DNA mediated by L1 retrotransposition in cancer genomes. Science.

[107] Scott EC, Gardner EJ, Masood A, Chuang NT, Vertino PM, Devine SE (2016). A hot L1 retrotransposon evades somatic repression and initiates human colorectal cancer. Genome Res.

[108] Tang Z, Steranka JP, Ma S, Grivainis M, Rodić N, Huang CRL (2017). Human transposon insertion profiling: analysis, visualization and identification of somatic LINE-1 insertions in ovarian cancer. Proc Natl Acad Sci U S A.

[109] Schauer SN, Carreira PE, Shukla R, Gerhardt DJ, Gerdes P, Sanchez-Luque FJ (2018). L1 retrotransposition is a common feature of mammalian hepatocarcinogenesis. Genome Res.

[110] Rodić N, Steranka JP, Makohon-Moore A, Moyer A, Shen P, Sharma R (2015). Retrotransposon insertions in the clonal evolution of pancreatic ductal adenocarcinoma. Nat Med.

[111] Burns KH (2017). Transposable elements in cancer. Nat Rev Cancer.

[112] Gorbunova V, Boeke JD, Helfand SL, Sedivy JM (2014). Sleeping dogs of the genome. Science.

[113] Feschotte C (2008). Transposable elements and the evolution of regulatory networks. Nat Rev Genet.

[114] Elbarbary RA, Lucas BA, Maquat LE (2016). Retrotransposons as regulators of gene expression. Science.

[115] Daniel C, Behm M, Öhman M (2015). The role of Alu elements in the cis-regulation of RNA processing. Cell Mol Life Sci.

[116] Cecco M, Criscione SW, Peckham EJ, Hillenmeyer S, Hamm EA, Manivannan J (2013). Genomes of replicatively senescent cells undergo global epigenetic changes leading to gene silencing and activation of transposable elements. Aging Cell.

[117] De Cecco M, Criscione SW, Peterson AL, Neretti N, Sedivy JM, Kreiling JA (2013). Transposable elements become active and mobile in the genomes of aging mammalian somatic tissues. Aging.

[118] Van Meter M, Kashyap M, Rezazadeh S, Geneva AJ, Morello TD, Seluanov A (2014). SIRT6 represses LINE1 retrotransposons by ribosylating KAP1 but this repression fails with stress and age. Nat Commun.

[119] Babaian A, Mager DL (2016). Endogenous retroviral promoter exaptation in human cancer. Mob DNA.

[120] Hedges DJ, Deininger PL (2007). Inviting instability: transposable elements, double-strand breaks, and the maintenance of genome integrity. Mutat Res Mol Mech Mutagen..

[121] Chiappinelli KB, Strissel PL, Desrichard A, Li H, Henke C, Akman B (2015). Inhibiting DNA methylation causes an interferon response in cancer via dsRNA including endogenous retroviruses. Cell.

[122] Kassiotis G, Stoye JP (2016). Immune responses to endogenous retroelements: taking the bad with the good. Nat Rev Immunol.

[123] Roulois D, Yau HL, Singhania R, Wang Y, Danesh A, Shen SY (2015). DNA-demethylating agents target colorectal cancer cells by inducing viral mimicry by endogenous transcripts. Cell.

[124] Crow YJ, Manel N (2015). Aicardi–Goutières syndrome and the type I interferonopathies. Nat Rev Immunol.

[125] Stetson DB, Ko JS, Heidmann T, Medzhitov R (2008). Trex1 prevents cell-intrinsic initiation of autoimmunity. Cell.

[126] Vargiu L, Rodriguez-Tomé P, Sperber GO, Cadeddu M, Grandi N, Blikstad V (2016). Classification and characterization of human endogenous retroviruses; mosaic forms are common. Retrovirology.

[127] Perron H, Jouvin-Marche E, Michel M, Ounanian-Paraz A, Camelo S, Dumon A (2001). Multiple sclerosis retrovirus particles and recombinant envelope trigger an abnormal immune response in vitro, by inducing polyclonal Vβ16 T-lymphocyte activation. Virology.

[128] Li W, Lee M-H, Henderson L, Tyagi R, Bachani M, Steiner J (2015). Human endogenous retrovirus-K contributes to motor neuron disease. Sci Transl Med.

[129] Downey RF, Sullivan FJ, Wang-Johanning F, Ambs S, Giles FJ, Glynn SA (2015). Human endogenous retrovirus K and cancer: innocent bystander or tumorigenic accomplice?. Int J Cancer.

[130] Kassiotis G, Stoye JP (2017). Making a virtue of necessity: the pleiotropic role of human endogenous retroviruses in cancer. Philos Trans R Soc Lond B Biol Sci.

[131] Jurka J, Kapitonov VV, Kohany O, Jurka MV (2007). Repetitive sequences in complex genomes: structure and evolution. Annu Rev Genomics Hum Genet.

[132] Naville M, Warren IA, Haftek-Terreau Z, Chalopin D, Brunet F, Levin P (2016). Not so bad after all: retroviruses and long terminal repeat retrotransposons as a source of new genes in vertebrates. Clin Microbiol Infect.

[133] Joly-Lopez Z, Bureau TE (2018). Exaptation of transposable element coding sequences. Curr Opin Genet Dev.

[134] Huang S, Tao X, Yuan S, Zhang Y, Li P, Beilinson HA (2016). Discovery of an active RAG transposon illuminates the origins of V(D)J recombination. Cell.

[135] Kapitonov VV, Koonin EV (2015). Evolution of the RAG1–RAG2 locus: both proteins came from the same transposon. Biol Direct.

[136] Frank JA, Feschotte C (2017). Co-option of endogenous viral sequences for host cell function. Curr Opin Virol.

[137] Cornelis G, Vernochet C, Carradec Q, Souquere S, Mulot B, Catzeflis F (2015). Retroviral envelope gene captures and syncytin exaptation for placentation in marsupials. Proc Natl Acad Sci U S A.

[138] Dupressoir A, Lavialle C, Heidmann T (2012). From ancestral infectious retroviruses to bona fide cellular genes: role of the captured syncytins in placentation. Placenta.

[139] Cornelis G, Funk M, Vernochet C, Leal F, Tarazona OA, Meurice G (2017). An endogenous retroviral envelope syncytin and its cognate receptor identified in the viviparous placental Mabuya lizard. Proc Natl Acad Sci U S A.

[140] Pastuzyn ED, Day CE, Kearns RB, Kyrke-Smith M, Taibi AV, McCormick J (2018). The neuronal gene arc encodes a repurposed retrotransposon Gag protein that mediates intercellular RNA transfer. Cell.

[141] Ashley J, Cordy B, Lucia D, Fradkin LG, Budnik V, Thomson T (2018). Retrovirus-like Gag protein Arc1 binds RNA and traffics across synaptic boutons. Cell.

[142] Lev-Maor G, Ram O, Kim E, Sela N, Goren A, Levanon EY (2008). Intronic Alus influence alternative splicing. PLoS Genet.

[143] Schmitz J, Brosius J (2011). Exonization of transposed elements: a challenge and opportunity for evolution. Biochimie.

[144] Richardson SR, Doucet AJ, Kopera HC, Moldovan JB, Garcia-Pérez JL, Moran JV (2015). The influence of LINE-1 and SINE retrotransposons on mammalian genomes. Microbiol Spectr.

[145] Xing J, Wang H, Belancio VP, Cordaux R, Deininger PL, Batzer MA (2006). Emergence of primate genes by retrotransposon-mediated sequence transduction. Proc Natl Acad Sci U S A.

[146] Esnault C, Maestre J, Heidmann T (2000). Human LINE retrotransposons generate processed pseudogenes. Nat Genet.

[147] Kubiak MR, Makalowska I (2017). Protein-coding genes’ retrocopies and their functions. Viruses.

[148] Carelli FN, Hayakawa T, Go Y, Imai H, Warnefors M, Kaessmann H (2016). The life history of retrocopies illuminates the evolution of new mammalian genes. Genome Res.

[149] Kapusta A, Kronenberg Z, Lynch VJ, Zhuo X, Ramsay L, Bourque G (2013). Transposable elements are major contributors to the origin, diversification, and regulation of vertebrate long noncoding RNAs. PLoS Genet.

[150] Lu X, Sachs F, Ramsay L, Jacques PE, Goke J, Bourque G (2014). The retrovirus HERVH is a long noncoding RNA required for human embryonic stem cell identity. Nat Struct Mol Biol.

[151] Wang J, Xie G, Singh M, Ghanbarian AT, Raskó T, Szvetnik A (2014). Primate-specific endogenous retrovirus-driven transcription defines naive-like stem cells. Nature.

[152] Durruthy-Durruthy J, Sebastiano V, Wossidlo M, Cepeda D, Cui J, Grow EJ (2015). The primate-specific noncoding RNA HPAT5 regulates pluripotency during human preimplantation development and nuclear reprogramming. Nat Genet.

[153] Percharde M, Lin C-J, Yin Y, Guan J, Peixoto GA, Bulut-Karslioglu A (2018). A LINE1-nucleolin partnership regulates early development and ESC identity. Cell.

[154] Jachowicz JW, Bing X, Pontabry J, Bošković A, Rando OJ, Torres-Padilla M-E (2017). LINE-1 activation after fertilization regulates global chromatin accessibility in the early mouse embryo. Nat Genet.

[155] Shen S, Lin L, Cai JJ, Jiang P, Kenkel EJ, Stroik MR (2011). Widespread establishment and regulatory impact of Alu exons in human genes. Proc Natl Acad Sci U S A.

[156] Johnson R, Guigó R (2014). The RIDL hypothesis: transposable elements as functional domains of long noncoding RNAs. RNA.

[157] Kelley DR, Hendrickson DG, Tenen D, Rinn JL (2014). Transposable elements modulate human RNA abundance and splicing via specific RNA-protein interactions. Genome Biol.

[158] Lubelsky Y, Ulitsky I (2018). Sequences enriched in Alu repeats drive nuclear localization of long RNAs in human cells. Nature.

[159] Piriyapongsa J, Mariño-Ramírez L, Jordan IK (2007). Origin and evolution of human microRNAs from transposable elements. Genetics.

[160] McCue AD, Slotkin RK (2012). Transposable element small RNAs as regulators of gene expression. Trends Genet.

[161] Bejerano G, Lowe CB, Ahituv N, King B, Siepel A, Salama SR (2006). A distal enhancer and an ultraconserved exon are derived from a novel retroposon. Nature.

[162] Chuong EB, Rumi MA, Soares MJ, Baker JC (2013). Endogenous retroviruses function as species-specific enhancer elements in the placenta. Nat Genet.

[163] Chuong EB, Elde NC, Feschotte C (2016). Regulatory evolution of innate immunity through co-option of endogenous retroviruses. Science.

[164] Trizzino M, Park Y, Holsbach-Beltrame M, Aracena K, Mika K, Caliskan M (2017). Transposable elements are the primary source of novelty in primate gene regulation. Genome Res.

[165] Thompson PJ, Macfarlan TS, Lorincz MC (2016). Long terminal repeats: from parasitic elements to building blocks of the transcriptional regulatory repertoire. Mol Cell.

[166] Jacques PE, Jeyakani J, Bourque G (2013). The majority of primate-specific regulatory sequences are derived from transposable elements. PLoS Genet.

[167] Wang T, Zeng J, Lowe CB, Sellers RG, Salama SR, Yang M (2007). Species-specific endogenous retroviruses shape the transcriptional network of the human tumor suppressor protein p53. Proc Natl Acad Sci U S A.

[168] Bourque G, Leong B, Vega VB, Chen X, Lee YL, Srinivasan KG (2008). Evolution of the mammalian transcription factor binding repertoire via transposable elements. Genome Res.

[169] Sundaram V, Cheng Y, Ma Z, Li D, Xing X, Edge P (2014). Widespread contribution of transposable elements to the innovation of gene regulatory networks. Genome Res.

[170] Ito J, Sugimoto R, Nakaoka H, Yamada S, Kimura T, Hayano T (2017). Systematic identification and characterization of regulatory elements derived from human endogenous retroviruses. PLOS Genet.

[171] Kunarso G, Chia NY, Jeyakani J, Hwang C, Lu X, Chan YS (2010). Transposable elements have rewired the core regulatory network of human embryonic stem cells. Nat Genet.

[172] Sun X, Wang X, Tang Z, Grivainis M, Kahler D, Yun C (2018). Transcription factor profiling reveals molecular choreography and key regulators of human retrotransposon expression. Proc Natl Acad Sci U S A.

[173] Schmidt D, Schwalie PC, Wilson MD, Ballester B, Goncalves A, Kutter C (2012). Waves of retrotransposon expansion remodel genome organization and CTCF binding in multiple mammalian lineages. Cell.

[174] Lunyak VV, Prefontaine GG, Nunez E, Cramer T, Ju BG, Ohgi KA (2007). Developmentally regulated activation of a SINE B2 repeat as a domain boundary in organogenesis. Science.

[175] Wang J, Vicente-García C, Seruggia D, Moltó E, Fernandez-Miñán A, Neto A (2015). MIR retrotransposon sequences provide insulators to the human genome. Proc Natl Acad Sci U S A.

[176] Lippman Z, Gendrel A-V, Black M, Vaughn MW, Dedhia N, McCombie WR (2004). Role of transposable elements in heterochromatin and epigenetic control. Nature.

[177] Rebollo R, Karimi MM, Bilenky M, Gagnier L, Miceli-Royer K, Zhang Y (2011). Retrotransposon-induced heterochromatin spreading in the mouse revealed by insertional polymorphisms. PLOS Genet.

[178] Chuong EB, Elde NC, Feschotte C (2017). Regulatory activities of transposable elements: from conflicts to benefits. Nat Rev Genet.

[179] Michaud EJ, Van Vugt MJ, Bultman SJ, Sweet HO, Davisson MT, Woychik RP (1994). Differential expression of a new dominant agouti allele (Aiapy) is correlated with methylation state and is influenced by parental lineage. Genes Dev.

[180] Morgan HD, Sutherland HGE, Martin DIK, Whitelaw E (1999). Epigenetic inheritance at the agouti locus in the mouse. Nat Genet.

[181] Ong-Abdullah M, Ordway JM, Jiang N, Ooi S-E, Kok S-Y, Sarpan N (2015). Loss of Karma transposon methylation underlies the mantled somaclonal variant of oil palm. Nature.

[182] Davidson EH, Britten RJ (1979). Regulation of gene expression: possible role of repetitive sequences. Science.

[183] Lynch VJ, Leclerc RD, May G, Wagner GP (2011). Transposon-mediated rewiring of gene regulatory networks contributed to the evolution of pregnancy in mammals. Nat Genet.

[184] Lynch VJ, Nnamani MC, Kapusta A, Brayer K, Plaza SL, Mazur EC (2015). Ancient transposable elements transformed the uterine regulatory landscape and transcriptome during the evolution of mammalian pregnancy. Cell Rep.

[185] Notwell JH, Chung T, Heavner W, Bejerano G (2015). A family of transposable elements co-opted into developmental enhancers in the mouse neocortex. Nat Commun.

[186] Makarevitch I, Waters AJ, West PT, Stitzer M, Hirsch CN, Ross-Ibarra J (2015). Transposable elements contribute to activation of maize genes in response to abiotic stress. PLoS Genet.

[187] Goerner-Potvin Patricia, Bourque Guillaume (2018). Computational tools to unmask transposable elements. Nature Reviews Genetics.

[188] Niu D, Wei H-J, Lin L, George H, Wang T, Lee I-H (2017). Inactivation of porcine endogenous retrovirus in pigs using CRISPR-Cas9. Science.

[189] Amabile A, Migliara A, Capasso P, Biffi M, Cittaro D, Naldini L (2016). Inheritable silencing of endogenous genes by hit-and-run targeted epigenetic editing. Cell.

[190] Guallar D, Bi X, Pardavila JA, Huang X, Saenz C, Shi X (2018). RNA-dependent chromatin targeting of TET2 for endogenous retrovirus control in pluripotent stem cells. Nat Genet.

[191] Fuentes DR, Swigut T, Wysocka J (2018). Systematic perturbation of retroviral LTRs reveals widespread long-range effects on human gene regulation. Elife.

[192] Langmead B, Trapnell C, Pop M, Salzberg SL (2009). Ultrafast and memory-efficient alignment of short DNA sequences to the human genome. Genome Biol.

[193] Wang J, Huda A, Lunyak VV, Jordan IK (2010). A Gibbs sampling strategy applied to the mapping of ambiguous short-sequence tags. Bioinformatics.

[194] Kahles A, Behr J, Rätsch G (2016). MMR: a tool for read multi-mapper resolution. Bioinformatics.

[195] ENCODE Project Consortium (2012). An integrated encyclopedia of DNA elements in the human genome. Nature.

[196] Graur D, Zheng Y, Price N, Azevedo RB, Zufall RA, Elhaik E (2013). On the immortality of television sets: “function” in the human genome according to the evolution-free gospel of ENCODE. Genome Biol Evol.

[197] Eddy SR (2013). The ENCODE project: missteps overshadowing a success. Curr Biol.

[198] Doolittle WF (2013). Is junk DNA bunk? A critique of ENCODE. Proc Natl Acad Sci U S A.

[199] Ohno S, Smith HH (1972). So much “junk” DNA in our genome. Evolution of genetic systems.

[200] Venuto D, Bourque G (2018). Identifying co-opted transposable elements using comparative epigenomics. Dev Growth Differ.

